# Optimization of reflectometry experiments using information theory

**DOI:** 10.1107/S1600576718017016

**Published:** 2019-02-01

**Authors:** Bradley W. Treece, Paul A. Kienzle, David P. Hoogerheide, Charles F. Majkrzak, Mathias Lösche, Frank Heinrich

**Affiliations:** a Department of Physics, Carnegie Mellon University, 5000 Forbes Avenue, Pittsburgh, Pennsylvania 15213, USA; b Center for Neutron Research, National Institute of Standards and Technology, 100 Bureau Drive, Gaithersburg, Maryland 20899-6102, USA; c Department of Biomedical Engineering, Carnegie Mellon University, 5000 Forbes Avenue, Pittsburgh, Pennsylvania 15213, USA

**Keywords:** neutron reflectometry, information content, experimental optimization

## Abstract

A framework based on Bayesian statistics and information theory is developed to optimize the design of surface-sensitive reflectometry experiments. The method applies to model-based reflectivity data analysis, uses simulated reflectivity data and is capable of optimizing experiments that probe a sample under more than one condition. After presentation of the underlying theory and its implementation, the framework is applied to exemplary test problems for which the information gain Δ*H* is determined. Reflectivity data are simulated for the current generation of neutron reflectometers at the NIST Center for Neutron Research. However, the simulation can be easily modified for X-ray or neutron instruments at any source. With application to structural biology in mind, this work explores the dependence of Δ*H* on the scattering length density of aqueous solutions in which the sample structure is bathed, on the counting time and on the maximum momentum transfer of the measurement. Finally, the impact of a buried magnetic reference layer on Δ*H* is investigated.

## Abstract 2

A framework for the optimization of neutron reflectometry experiments based on Bayesian statistics and information theory is presented.

## 1. Introduction   

Neutron reflectometry (NR) is a structure determination technique that resolves the thickness and composition of thin films at interfaces and surfaces with near-ångström resolution (Smith & Majkrzak, 2006 ▸). Applications of NR reach from hard-condensed matter (Majkrzak *et al.*, 2006 ▸) to soft matter (Russell, 1990 ▸), including structural biology of lipid membranes (Heinrich & Lösche, 2014 ▸). Given the limited availability of neutrons for scattering experiments and the flexibility in isotopic labeling of distinct components of the surface structure, it is worthwhile to optimize the experimental design with respect to the information gain. Presently, the design of neutron scattering experiments mostly follows rules of thumb, *i.e.* experience gained in similar experiments in the past. Here, we implement a quantitative and predictive framework to plan reflectometry work based on rigorous estimates of the information gained in a particular implementation of the experiment. With minor changes, this framework is applicable to X-ray reflectometry and, with some extension, to neutron and X-ray small-angle scattering.

In the recent past, Bayesian statistical methods have found applications in reflectometry for robust global model fitting and the determination of confidence limits on model parameters (Sivia & Webster, 1998 ▸; Kirby *et al.*, 2012 ▸; Maranville *et al.*, 2016 ▸; Lesniewski *et al.*, 2016 ▸). In particular, the work of Sivia and co-workers has provided a solid foundation for the application of Bayesian statistics to reflectivity data, discussing aspects such as parameter estimation, model selection and experimental design. Our work concerning experimental optimization adds to this foundation by introducing model fitting based on a Monte Carlo Markov chain (MCMC) simulation, which by design yields a sample of the posterior parameter density function (PDF) (Yustres *et al.*, 2012 ▸; Braak & Vrugt, 2008 ▸; Lesniewski *et al.*, 2016 ▸). A measure of the information gain from a given experiment is obtained from comparing the entropies of the posterior and prior PDFs, which represent the knowledge about the sample after and before the experiment, respectively. We show that with these two additions a flexible numerical framework for experimental design can be built. In developing this methodology for reflectometry, we closely follow established implementations in other fields such as systems biology (Liepe *et al.*, 2013 ▸, 2014 ▸).

**Figure 1:**
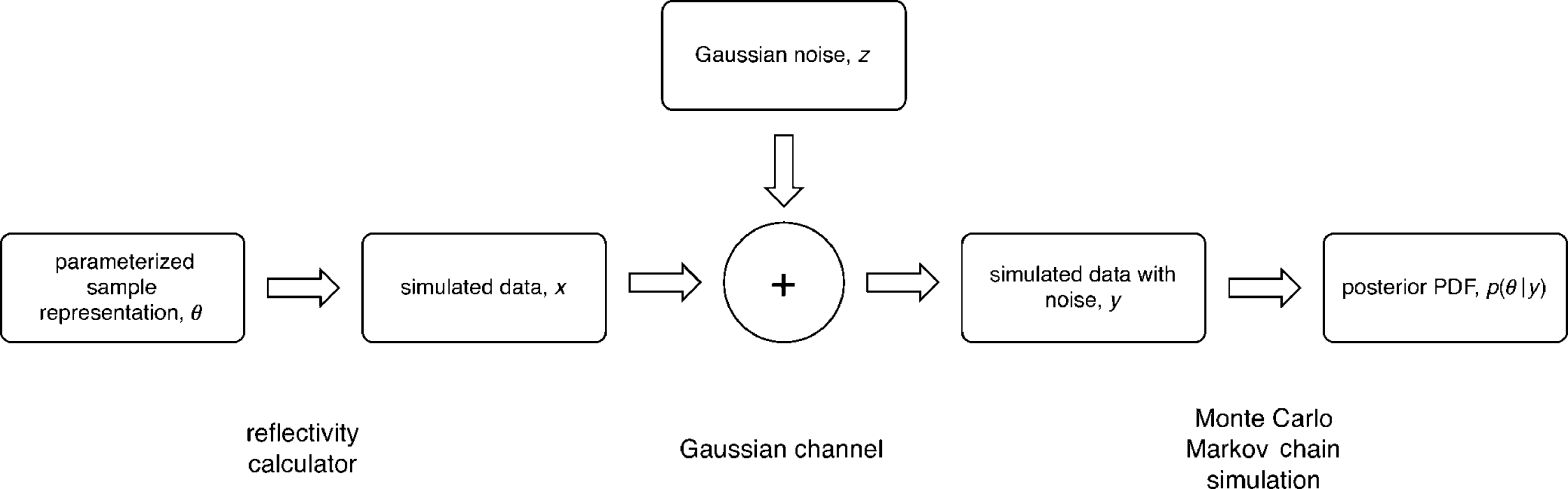
Information processing steps in a virtual reflectivity experiment. The information gain is the difference in entropy between the posterior and prior PDFs.

Fig. 1 ▸ summarizes the implemented method to quantify the information gain of an experiment. We start with a model-dependent description of a hypothetical sample structure *S* and instrument configuration *E* parameterized by a vector θ ∈ Θ. (Capital letters denote a random variable, and lower-case letters denote a particular instance of a random variable.) Importantly, θ is not randomly drawn from Θ according to the prior PDF, as we do not optimize over different sample configurations within the prior. Using a model *X*_*S*,*E*_(θ), noise-free reflectivity data *X*_*S*,*E*_(θ) → *x*(*Q*_*z*_) are simulated over a finite range of discrete, experimentally accessible momentum transfer values *Q*_*z*_. Random normal noise *z*(*Q*_*z*_) is added to *x*(*Q*_*z*_) to obtain simulated sets of noisy data *y*(*Q*_*z*_) that could have occurred in a real measurement of the hypothetical structure. The standard deviation σ(*Q*_*z*_) of the normal noise *Z* depends on the instrument configuration, the momentum transfer *Q*_*z*_ and the value of *x*(*Q*_*z*_) itself. It generally differs for every data point. Finally, model parameters are retrieved from *y* using an MCMC simulation that returns a sample of the posterior PDF *p*(θ | *y*). The information gain of the virtual experiment is evaluated as the difference in entropy of the prior and posterior PDFs, 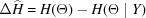. Since the MCMC simulation employs the same model that was used to calculate 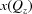, 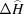 is a measure of the gain in information exclusively about model and experimental parameters contained in Θ. Other parameters intrinsic to the model that have fixed values, such as the known scattering length density of a substrate supporting the interfacial structure and instrumental parameters like those defining the resolution function, do not affect 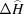. To optimize an NR experiment, sample or experimental properties are systematically varied to determine the maximum 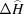 in the search space.

Different approaches to determine the information content for small-angle scattering data have been established in the past (Moore, 1980 ▸; Taupin & Luzzati, 1982 ▸; Luzzati & Taupin, 1986 ▸; Müller *et al.*, 1996 ▸; Vestergaard & Hansen, 2006 ▸; Pedersen *et al.*, 2014 ▸; Konarev & Svergun, 2015 ▸; Larsen *et al.*, 2018 ▸). While the employed methods differ substantially, these approaches have in common that the information content of the experiment is quantified either directly from *y*, or from *y* given *x* and θ. Our method is strictly model based and it describes the information gain from virtual experiments using a series of discrete information processing steps as shown in Fig. 1 ▸, ultimately comparing the two endpoints of this process with respect to the information content. This has the advantage that it separates the information gain on model fit parameters from known model and experimental parameters, about which information is also carried by *x* and *y*. This procedure has a substantial advantage over other implementations in that it can handle information distributed across multiple related measurements that are analyzed simultaneously with one model.

After establishing the methodology, we apply it to a set of simple model systems, thereby demonstrating the optimization of fundamental experimental properties, such as counting time, maximum momentum transfer and the choice of the scattering length density (SLD) of the bulk solvent in NR measurements of fluid-immersed samples. These examples have been chosen to best illustrate the broad usefulness of the technique, and they can easily be extended to encompass other experimental situations of practical interest. For example, while the method described here is applied to the current generation of monochromatic neutron reflectometers, it can be adapted to other types of reflectometers or be used to predict the performance of experimental stations under development.

## 2. Theory and implementation   

### 2.1. Information content of specular reflection data   

A specular neutron or X-ray reflectometry experiment on a sample *S* using an experimental configuration *E* results in a particular measurement of the data, *y* ∈ *Y*. Experimental results are generally analyzed in terms of a model, *X*_*S*,*E*_(θ), that relates a model parameter vector θ ∈ Θ to an expected experimental outcome 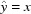. The aim of data analysis is to find the posterior PDF *p*(θ | *y*) by which a particular parameter vector θ is realized given *y* and *X*_*S*,*E*_(θ). The traditional task of finding the vector θ that produces the best fit to the data, or the maximum of *p*(θ | *y*), is therefore only a particular aspect within this broader definition of data analysis.

The information gain Δ*H* is defined as the difference between the entropy *H*(Θ) of the prior PDF *p*(θ), representing the knowledge before the experiment, and the entropy *H*(Θ | *y*) of the posterior PDF *p*(θ | *y*), obtained after the measurement yielded a particular experimental outcome *y* ∈ *Y*: 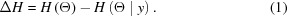
Both entropies are functionals of their respective continuous PDFs: 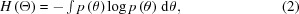


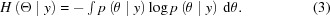
(All logarithms in this work are taken to the base of two, such that entropies are calculated in bits.) This approach does not consider that the experimental outcome *y* is a random variable itself, drawn from a pool of possible experimental outcomes *Y*. The appropriate, but significantly more expensive, quantity to calculate is the expected information gain 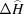 given all possible experimental outcomes *y*, which equals the mutual information *I* between the random variables *Y* and Θ: 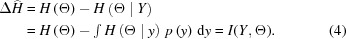
Using equation (4), 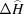 can in principle be computed as a Monte Carlo integration over Θ and *Y* (Liepe *et al.*, 2013 ▸). The prior predictive distribution *p*(*y*) additionally needs to be computed (Liepe *et al.*, 2013 ▸), and it can be expressed in terms of the prior and posterior PDFs using Bayes’ theorem: 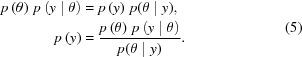
The conditional PDF *p*(*y* | θ) of observing a particular experimental outcome *y*, given a parameter vector θ, can be obtained using the model *X*_*S*,*E*_(θ) and instrument-specific normal variate random noise on *n* data points of *y*(*Q*_*z*_) with a standard deviation σ(*Q*_*z*_):
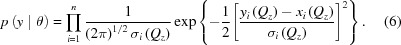
In practice, however, such a nested Monte Carlo integration is computationally costly.^1^ We therefore approximate 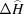 from the average of up to ten calculations of Δ*H* using independently simulated experimental data *y* (Liepe *et al.*, 2013 ▸).

### 2.2. Implementation of the algorithm   

#### 2.2.1. Simulation of experimental data   

This work is carried out with simulated data to avoid systematic errors due to particular experimental instrumentation and to explore a large parameter space for optimization. Experimental data *y*(*Q*_*z*_) are simulated for the Magik reflectometer at the NIST Center for Neutron Research (NCNR) in Gaithersburg, MD, USA (Dura *et al.*, 2006 ▸), with a beam footprint on the sample surface of 2.5 × 5 cm, equipped with a fluids cell for solvent-immersed samples. The models *X*_*S*,*E*_(θ) of interfacial structures were implementations of stratified slabs of homogeneous SLD (slab models) (Ankner & Majkrzak, 1992 ▸). Noise-free experimental outcomes *x*(*Q*_*z*_) were calculated with *refl1d* (Kirby *et al.*, 2012 ▸). To obtain the final simulated reflectivity *y*(*Q*_*z*_), normally distributed random noise *z*(*Q*_*z*_) with standard deviations σ_*S*,*E*_[*Q*_*z*_, *x*(*Q*_*z*_), θ] was added to *x*(*Q*_*z*_). A detailed description of the data simulation is provided in the supporting information.

For the optimization of a particular experiment, parameter vectors θ ∈ Θ for data simulation should be strictly drawn at random from Θ according to the prior PDF *p*(θ) (see *Introduction* [Sec sec1]). However, for the problems discussed in this work, and for many other applications, the sample structure is sufficiently well known that variations within *p*(θ) are not expected to significantly affect Δ*H* and are negligible compared with changes in Δ*H* that occur when systematically varying parameters over much larger ranges during the experimental optimization. Choosing one sample representation θ ∈ Θ from the prior PDF has the additional advantage that a costly Monte Carlo simulation over the prior PDF, which would be otherwise necessary, is avoided (Liepe *et al.*, 2013 ▸). For largely unknown sample structures that would be implemented by a much wider prior PDF, the dependence of Δ*H* on the particular sample representation θ ∈ Θ might not be negligible. In this case, the framework presented in this work can easily be extended to include a Monte Carlo simulation over the prior PDF.

#### 2.2.2. Entropy of the prior parameter density function   

Lacking more detailed prior knowledge, the prior PDF *p*(θ) is the product of the prior probabilities of the assumed independent vector components θ_*i*_. It is further assumed that the PDF of each component, *p*(θ_*i*_), is constant over an interval Δθ_*i*_ with *p*(θ_*i*_) = 1/Δθ_*i*_:
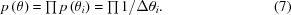
Using equation (2), the entropy of *p*(θ) becomes the sum of the entropies of the independent *p*(θ_*i*_): 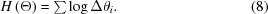
The prior PDF is subjectively set by the experimenter. In addition to the calculation of Δ*H*, it is used to compute the acceptance probability of new states of the Markov chain during the MCMC analysis of *y*(*Q*_*z*_). As such, the choice of Δθ_*i*_ affects the posterior PDF in that it excludes parameter values outside of Δθ_*i*_. Only parameters with non-uniform contributions to the posterior PDF, or in other words parameters that can be resolved with respect to the prior PDF, add to the information gain (see Section 3.1.3 for a detailed discussion). A change in interval length Δθ_*i*_ for a resolvable parameter leads to a constant offset in *H*(Θ) and, therefore, Δ*H*, which is inconsequential for the purpose of experimental optimization, as it relies on relative differences in Δ*H*.

#### 2.2.3. Entropy of the posterior parameter density function   

The posterior PDF is obtained from an MCMC simulation implemented in *refl1d* (Kirby *et al.*, 2012 ▸) using the simulated data *y* and the model *X*_*S*,*E*_(θ) as inputs. MCMC analysis yields an unnormalized sample of the posterior PDF. We calculated the entropy of *p*(θ | *y*) using two different methods. The first method constructs a multivariate normal (MVN) probability density approximation from a random sample of 1000 points of the posterior:
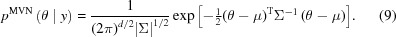
The vector μ is the mean of the sample of *d*-dimensional parameter vectors θ. |Σ| denotes the determinant of the variance–covariance matrix Σ of θ. Both values can be defined in terms of an expectation value 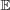:
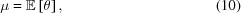


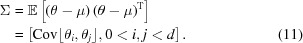
The entropy of the MVN distribution is then computed as (Chen *et al.*, 2016 ▸)
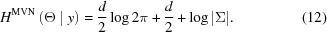



The second method to calculate the entropy of the posterior follows Kramer *et al.* (2010 ▸). Here, the entropy of the posterior is obtained by Monte Carlo sampling from the unnormalized MCMC output using a sample size of 5000, while the normalization factor is obtained from a kernel density estimate using a Gaussian kernel (Silverman, 1986 ▸). In the following, we denote this approach as the KDN method.

The sample sizes for the MVN and KDN approaches represent limits for which a computation of an equilibrated MCMC plus entropy estimate remains feasible given current computational resources. Because of those limits on sample size and the high dimensionality of NR models, an accurate and robust computation of the posterior entropy is often challenging. The MVN estimate fulfills here the role of a widely used reference against which the KDN estimate can be validated. As shown in Section 3, the MVN approximation was robust over repetitions of MCMC simulations but tended to underestimate the entropy of the posterior and, therefore, overestimated Δ*H*. This is not unexpected, as the MVN approximation performs less well on non-normal, *i.e.* asymmetric and heavily tailed, distributions (Kramer *et al.*, 2010 ▸). The KDN method proved less robust, in turn, leading to somewhat larger standard deviations on Δ*H* and occasional outliers that were identified and eliminated.

#### 2.2.4. Information gain   

As discussed above, the computation of 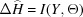 is significantly costlier than calculating the gain in information Δ*H* obtained from only a single experimental representation *y*. For large multivariate models such as those used in NR, computing 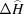 is currently not feasible. The differences between Δ*H* and 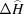 have been determined for models with fewer parameters. While significant, they were shown to be smaller than the changes in entropy due to experimental optimization in other applications (Liepe *et al.*, 2013 ▸). This observation is in agreement with results in this work. We estimated the difference between 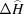 and Δ*H* by averaging multiple independent values of Δ*H* and showed that the variations in Δ*H* for individual points of the optimization are significantly smaller than the changes in Δ*H* over the entire range of systematically varied parameters to be optimized.

#### 2.2.5. Marginalization of the posterior parameter density function   

Most models *X*_*S*,*E*_ contain a subset of nuisance parameters δ that are required for constructing a valid model but are not of practical interest to the experimenter. Together with the parameters of interest η, they form the parameter space θ = (η, δ). Consequently, the relevant quantity for optimizing an NR experiment is often the marginal entropy of the posterior *H*_η_(Θ | *y*) with respect to the parameters of interest (Sivia & Webster, 1998 ▸; Chen *et al.*, 2016 ▸): 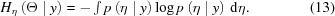
The marginal posterior PDF *p*(η | *y*) is obtained by integrating the joint probability of η and δ over the nuisance parameters δ: 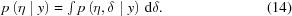
Using an MVN distribution, a marginal entropy *H*_η_^MVN^(Θ | *y*) is calculated rather easily by dropping the unwanted parameters from the covariance matrix and the mean vector: 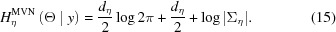
The computation of a KDN equivalent of the marginal entropy that involves Monte Carlo sampling from the MCMC-obtained posterior PDF is difficult and will be the topic of a future study. In this work, we exclusively compute total entropies of the posterior PDF and evaluate confidence limits on parameters of interest separately for selected points in the optimization space.

## 3. Results   

### 3.1. A test structure   

**Figure 2:**
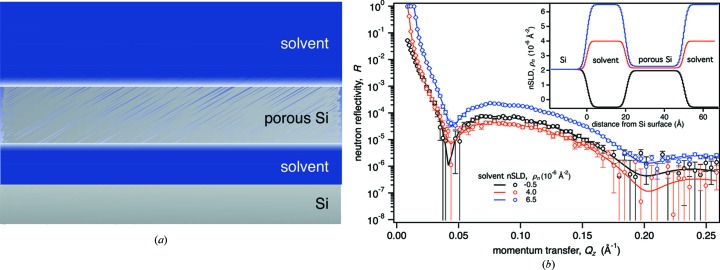
(*a*) Structural model of the test system. A 30 Å-thick porous Si layer (95% Si by volume) is surrounded by aqueous solvent and suspended at a distance of 20 Å from a solid Si surface. Pores in the Si layer are solvent filled. (*b*) Calculated reflectivities with simulated noise for three different solvent SLD values, reflectivity curves that are best fits to the data and their associated SLD profiles (inset). The noisy data and error bars correspond to those expected in a measurement of this hypothetical sample at a current-generation reactor-based instrument such as the Magik neutron reflectometer at the NCNR. Error bars represent 68% confidence limits.

For a first demonstration of the method, we start with a simple artificial interfacial structure: a porous, atomically flat Si layer suspended above a planar solid Si substrate in aqueous solvent (Fig. 2 ▸). This structure is characterized at first with one and then with two NR measurements, evaluated with a simple slab model, and analyzed for the resulting information gain under systematic variation of the SLD of the solvent, *ρ_n_*. Model parameters are provided in Table 1 ▸. The aim of the optimization is to identify the isotopic constitution of the aqueous solvent that maximizes information gain as *ρ_n_* is varied between that of H_2_O (ρ_*n*_ ≃ −0.5 × 10^−6^ Å^−2^) and D_2_O (ρ_*n*_ ≃ 6.5 × 10^−6^ Å^−2^).

#### 3.1.1. One solvent contrast   

**Figure 3:**
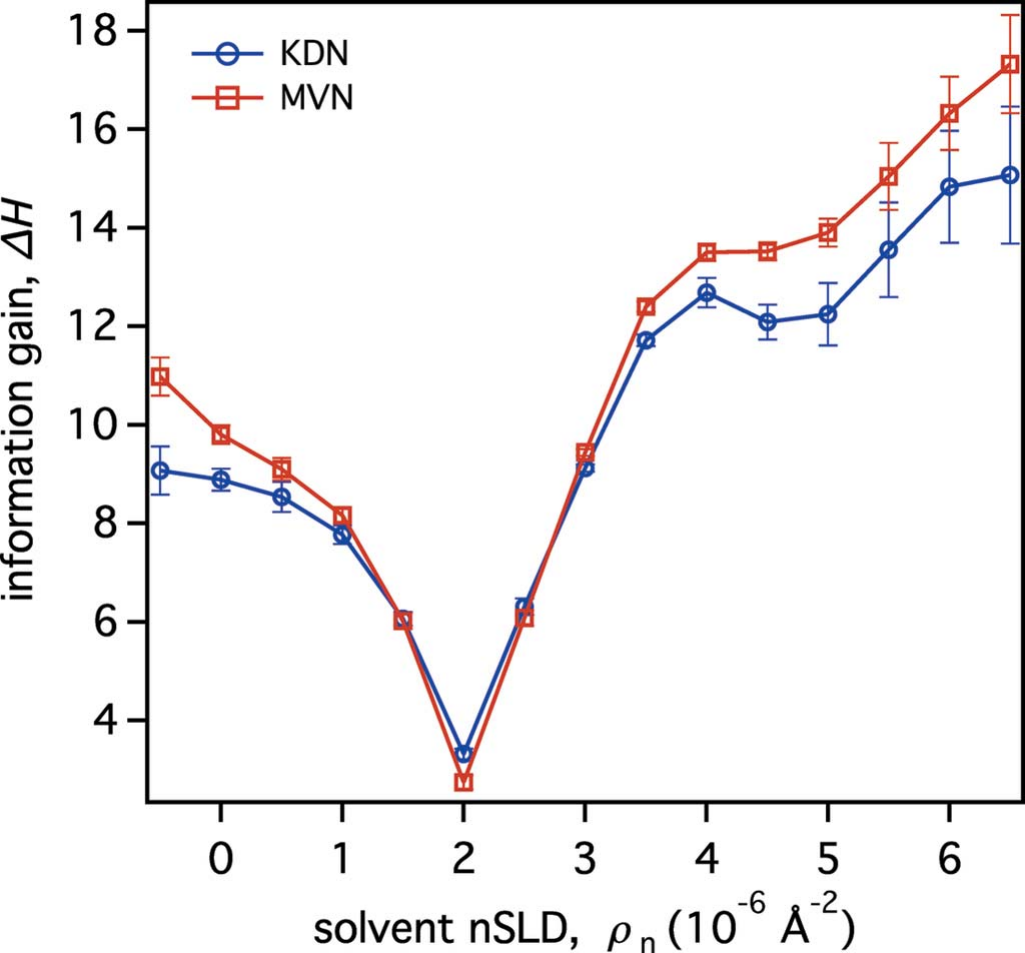
Information gain Δ*H* as a function of the SLD of the aqueous solvent in a virtual NR experiment of the model structure shown in Fig. 2 ▸. Δ*H* calculated using the MVN and KDN approximations follow each other closely. Error bars indicate one standard deviation obtained from five independent simulations.

Fig. 3 ▸ shows the expected information gain from a single NR measurement as a function of the SLD of the aqueous medium that surrounds and penetrates the porous Si layer (‘one solvent contrast’). The MVN and KDN methods for entropy determination yield similar results, with the MVN results consistently slightly higher than the KDN results. The error bars in Fig. 3 ▸ represent standard deviations from five independent simulations per data point and allow us to assess the error introduced by computing Δ*H* instead of 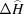. For this particular example, this error is significantly smaller than the changes in Δ*H* due to variation in solvent SLD.

The minimum information gain is observed under the condition that the bulk solvent SLD matches that of Si (ρ_*n*_ ≃ 2 × 10^−6^ Å^−2^). In this case, the porous solvent-filled Si slab, the substrate and the solvent all have the same SLD and are thus ‘invisible’ to neutrons. The residual gain at the minimum of the curve, Δ*H* ≃ 3 bits, stems from a high confidence in determining the SLDs of the porous silicon layer, the bulk solvent and the scattering background. Because the SLD of the semi-infinite Si substrate is known, the unknown SLDs are well determined under matching conditions, even though the observed neutron reflectivity is zero. Other model parameters can only be resolved if the experimenter uses a solvent with an SLD different from that of Si. Consequently, the gain in information increases when its SLD deviates from that of Si, reaching Δ*H* ≃ 10 bits for H_2_O-based solvent and Δ*H* ≃ 15 bits for D_2_O. For the same absolute difference of the solvent SLD from that of Si, positive SLD deviations yield a larger information gain than negative deviations. This phenomenon is due to several effects. First, because the incoherent scattering from D_2_O is lower than that from H_2_O, samples bathed in D_2_O-rich solvent show lower intrinsic scattering background. Second, the higher SLDs of D_2_O-rich solvents lead to higher neutron reflectivity throughout the simulated range of *Q*_*z*_ (0.08 ≤ *Q*_*z*_ ≤ 0.26 Å^−1^), which can be determined with higher confidence. Finally, the presence of a critical angle of total internal reflection in the NR curve increases the gain in information for solvent SLDs with ρ_*n*_ > 4 × 10^−6^ Å^−2^, at which this critical angle can be observed within the simulated *Q*_*z*_ range.

Explicit fit parameters and their uncertainties for three exemplary bulk solvent SLD values are listed in Table 1 ▸. The parameter uncertainties for ρ_*n*_ = −0.5 × 10^−6^ Å^−2^ and ρ_*n*_ = 6.5 × 10^−6^ Å^−2^ are significantly smaller over the entire set of model parameters, reflecting the increased information gain for those solvents.

To put the abstract values of Δ*H* given in Fig. 3 ▸ in perspective, the following simplified comparison is educative. Under the assumption that the posterior PDF of a single uncorrelated parameter can be approximated by a Gaussian distribution, the contribution of this parameter to the posterior entropy is determined by the standard deviation σ of the distribution: 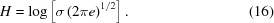
With respect to the entropy of the corresponding uniform prior PDF over the interval Δθ [equation (8)], the contribution to the information gain from this single parameter is
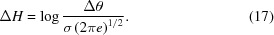
A Gaussian posterior PDF with a standard deviation approximately one-quarter [*i.e.* 1/(2π*e*)^1/2^] of the width of a uniform prior PDF has the same entropy as the latter and yields therefore zero information gain. Standard deviations above this threshold contribute a limited loss of information, particularly for the MVN estimate, only because of the different functional forms used to describe the prior and posterior PDFs. Equations (8) and (16) also show that decreasing the widths of either a uniform prior or a Gaussian posterior PDF by one-half changes the information gain by ∼1 bit (while neglecting parameter correlations).

#### 3.1.2. Two solvent contrasts   

**Figure 4:**
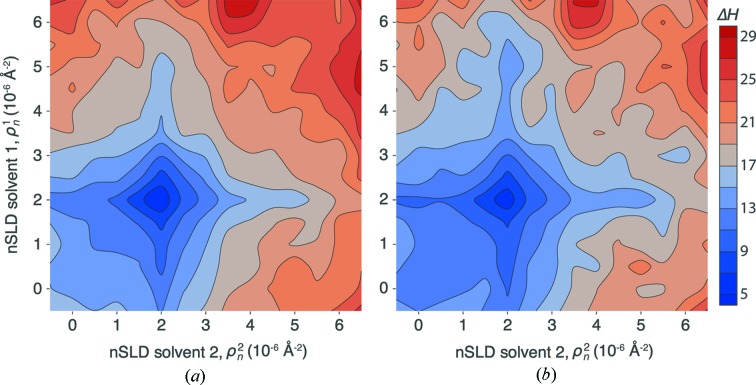
Information gain Δ*H* as a function of the aqueous solvent SLDs in two simultaneously evaluated, independent NR measurements of the model structure in Fig. 2 ▸. SLDs are varied in steps of 0.5 × 10^−6^ Å^−2^. Entropies of the posterior were calculated using the MVN (*a*) and KDN (*b*) approximations. Symmetry-related data were independently calculated with both methods to obtain a visual impression of data reproducibility.

The information gain of an NR experiment that consists of two reflectivity measurements with different solvents is shown in Fig. 4 ▸. The two solvent SLDs in these measurements were independently varied between those of pure H_2_O and D_2_O, and data analysis was performed under the constraint that both structural models share the same set of parameters, except for the two solvent SLDs and their associated background levels. The minimum information gain is observed when both solvent SLDs are ρ_*n*_ = 2 × 10^−6^ Å^−2^. Particularly high information gains are found for bulk compositions at the extreme margins of solvent SLDs, (ρ_*n*_^1^, ρ_*n*_^2^) = (−0.5 × 10^−6^ Å^−2^, 6.5 × 10^−6^ Å^−2^). However, a combination of measurements with pure D_2_O and an H_2_O/D_2_O mixture with ρ_*n*_ = 4 × 10^−6^ Å^−2^ (denoted as CM4) yields a comparable information gain.

Similarly to the previous optimization, the KDN and MVN entropy estimates in Fig. 4 ▸ yield qualitatively equivalent results. While the MVN estimate slightly overestimates the information gain, the KDN estimate shows somewhat larger uncertainties in each data point. In all further examples discussed in this work, we only used KDN estimates.

Fit parameters and their uncertainties for three regions of particularly small and large Δ*H* (Fig. 4 ▸) are provided in Table 2 ▸. For the minimum information gain at (ρ_*n*_^1^, ρ_*n*_^2^) = (2.0 × 10^−6^ Å^−2^, 2.0 × 10^−6^ Å^−2^), the result is comparable to the experiment with one solvent contrast. Only the parameter uncertainties for the SLD and volume fraction of the porous Si, and the SLDs of both bulk solvents, show significant improvement over the prior PDFs. The parameter uncertainties for (−0.5 × 10^−6^ Å^−2^, 6.5 × 10^−6^ Å^−2^) and (6.5 × 10^−6^ Å^−2^, 4 × 10^−6^ Å^−2^) are significantly smaller than for (2.0 × 10^−6^ Å^−2^, 2.0 × 10^−6^ Å^−2^) over the entire set of model parameters.

Evaluations such as those shown in Fig. 4 ▸ are invaluable to determine whether it is advantageous in a real experiment to consecutively measure two NR curves from a sample bathed in distinct solvents or, rather, to allocate the same total measurement time to a single measurement with one solvent. In this example, values at the plot diagonal are consistently lower than off-diagonal values, which would argue in favor of conducting two distinct measurements.

#### 3.1.3. Dependence of Δ*H* on counting time   

**Figure 5:**
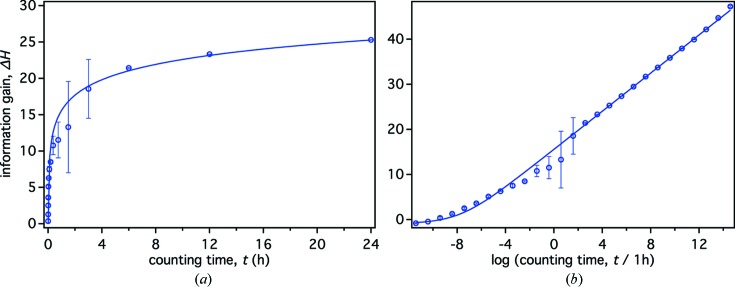
Information gain Δ*H* as a function of the counting time *t* for a single NR measurement of the model structure (Fig. 2 ▸) in D_2_O, calculated using the KDN estimate and plotted on linear (*a*) and logarithmic (*b*) time scales. The continuous curves are fits to equation (23), which describes the information gain as being limited by the capacity of *m*′ parallel independent Gaussian channels. The displayed time range in (*a*) has been shortened to 24 h to focus on experimentally relevant measurement times. Error bars indicate one standard deviation obtained from ten independent simulations.

Fig. 5 ▸(*a*) shows the information gain Δ*H* as a function of the counting time *t* for a single NR measurement of the model structure (Fig. 2 ▸) immersed in D_2_O. After an initial fast increase of Δ*H* within the first 3 h of measurement, the information gain quickly enters a region of diminishing return in which further improvement requires increasingly long counting times. For the presented example and simulated instrument, we consider the transition region between those two regimes at 3–6 h an optimal range of counting time. The observed time dependence of the information gain is similar to those previously reported (Pedersen *et al.*, 2014 ▸; Berk & Majkrzak, 2009 ▸). It suggests that, at least for this simple test structure, an increase in neutron flux on future instrumentation will allow for shorter measurement times but may not yield significantly reduced fit parameter confidence limits.

To describe the functional form of the relation shown in Fig. 5 ▸, we simplify the situation and consider the capacity of the Gaussian channel shown in Fig. 1 ▸ to be the single limiting factor for the information gain. In other words, for the current example we assume that the exact parameter vector θ used for data simulation could be retrieved by the MCMC simulation given noise-free reflectivity data *y*(*Q*_*z*_). We therefore neglect other limiting factors such as the loss of phase information during reflectivity simulation or shortcomings of the MCMC algorithm. The capacity *C* of a communication channel is defined as the maximum mutual information between the input *X* and the output *Y* for all possible choices of *p*(*x*). In communication theory, *C* sets the maximum transmission rate of information over the channel; in our example it constitutes an upper limit on the information gain on *x* by knowing *y*.

As shown in Fig. 1 ▸, the Gaussian channel adds random normal noise *z*(*Q*_*z*_) with standard deviation σ(*Q*_*z*_) to the noise-free simulated reflectivity *x*(*Q*_*z*_), thus providing the reflectivity with noise *y*(*Q*_*z*_). Since the reflectivity is simulated for *n* discrete values of *Q*_*z*_ (*n* data points), such a Gaussian channel can be described by the combined action of *n* independent parallel Gaussian channels, each of which adds random normal noise to one data point of the simulated reflectivity. *n* independent parallel Gaussian channels have a combined maximum capacity *C* that depends on the signal-to-noise ratio *y*/σ per channel (Cover & Thomas, 2006 ▸): 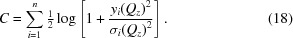



Neglecting contributions from background subtraction and incident intensity normalization, the signal-to-noise ratio *y*/σ can be computed solely from counting statistics. For each reflectivity data point, *y*/σ depends only on the number of specular counts *N*, which is the product of the counting time *t* and a constant specular count rate *r*: 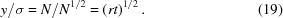
We can therefore rewrite the combined capacity of the Gaussian channels as
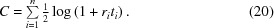
In a typical NR measurement, the signal-to-noise ratio *y*/σ = (*rt*)^1/2^ is kept approximately constant over the entire *Q*_*z*_ range by increasing both counting time and beam cross section as *Q*_*z*_ increases, in order to offset the general *Q*_*z*_^−4^ dependence of the specular reflectivity. We therefore simplify equation (20) by assuming that all channels have the same relative variance, which is measured in an effective time *t* and at an effective rate *r*. We arbitrarily choose that *t* represents the total counting time of the reflectivity curve (instead of, for example, choosing the average measurement time per point which would only change the effective rate).^2^ On the basis of the Shannon–Nyquist sampling theorem (Pedersen *et al.*, 2014 ▸; Konarev & Svergun, 2015 ▸), the reflectivity data in the example are heavily oversampled. In addition, *R* is band limited. Consequently, not all *n* channels are independent, and we can write the channel capacity as that of *m* effective independent channels (*m* < *n*) (Cover & Thomas, 2006 ▸): 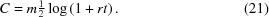
The channel capacity *C* imposes an upper limit on the actual channel rate *I*(*X*, *Y*), which itself is an upper limit on the information gain Δ*H* of the entire virtual experiment shown in Fig. 1 ▸: 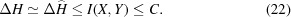
Consequently, we apply the following equation for analysis of the KDN-derived information gain (Fig. 5 ▸): 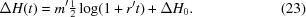
The coefficient *m*′ can be interpreted as the number of independent parameters determined in the experiment, and *r*′ is associated with an average rate of increase in information gain per parameter. Fig. 5 ▸ shows the fit to Δ*H*(*t*) that yields *m*′ = 4.2 ± 0.1 and *r*′ = 219 ± 5 h^−1^. The value of *m*′ indicates four independent parameters, and inspection of the last column in Table 1 ▸ confirms this estimate, as four out of seven parameters show a significant improvement over the prior PDF (*t* = 6 h). Δ*H*_0_ is the systematic error in calculating the information gain for *t* → 0, which stems from evaluating a uniform posterior PDF that equals the prior PDF using the KDN estimate (see Section 3.1.1). Δ*H*_0_ was determined to be −0.89 ± 0.05 bits.

The values of Δ*H* for 0.375 ≤ *t* ≤ 3 h show a comparatively high uncertainty (see Fig. 5 ▸). When inspecting individual parameter uncertainties over this interval (data not shown), it was found that in this region a transition occurs, in which the number of independent parameters that can be resolved (are in scope of the prior PDF) increases from three to four, and variations in the simulated reflectivity due to random noise can lead to either outcome in the MCMC analysis. Accordingly, when fitting Δ*H*(*t*) using equation (23) and a limited time interval 0 ≤ *t* < 0.375 h, a coefficient *m*′ = 3.0 ± 0.1 is obtained (fit not shown), which agrees with the ability to resolve three independent parameters. This indicates that, strictly, Δ*H*(*t*) has to be fitted piecewise over intervals of *t*, in which the number of resolvable independent parameters does not change. A thorough exploration of this aspect goes beyond the objective of this work and is left for a future study.

#### 3.1.4. Dependence of Δ*H* on the maximum momentum transfer   

**Figure 6:**
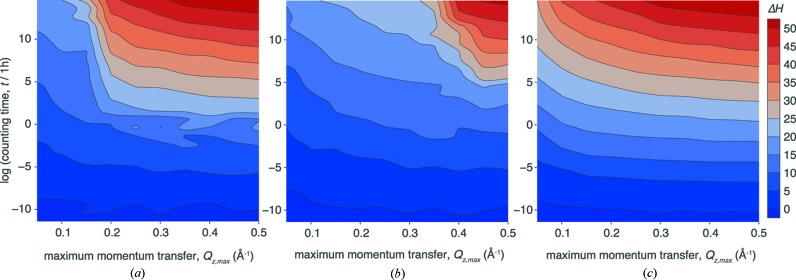
(*a*) Information gain (KDN estimate) as a function of maximum momentum transfer *Q*_*z*,max_ and counting time *t* of the measurement for the model structure shown in Fig. 2 ▸ and related structures in which all layer thicknesses were multiplied by 1/2 (*b*) and 2 (*c*). The counting times shown apply to *Q*_*z*,max_ = 0.26 Å^−1^, but were shorter and longer for smaller and larger *Q*_*z*,max_, respectively, as we have chosen an optimization scheme that preserves the counting statistics for individual data points, but not the total counting time when varying *Q*_*z*,max_.

Fig. 6 ▸ shows the dependence of the information gain on counting time *t* and maximum momentum transfer *Q*_*z*,max_ of the simulated data for the test structure (Fig. 2 ▸) immersed in D_2_O [Fig. 6 ▸(*a*)], and two related structures in which all layer thicknesses are scaled by a factor of 0.5 [Fig. 6 ▸(*b*)] or a factor of 2 [Fig. 6 ▸(*c*)]. For all three structures, Δ*H* shows an increase with *t* similar to that in Fig. 5 ▸, which is equivalent to a vertical slice of the independent optimization shown in Fig. 6 ▸(*a*) at *Q*_*z*,max_ = 0.26 Å^−1^. All structures show a rather sudden increase in information gain when the reflectivity is extended beyond a certain critical value of *Q*_*z*,max_, which roughly matches the position of the second minimum of the reflectivity curves [Fig. 2 ▸(*b*)]. For the original test structure, this transition occurs at *Q*_*z*,max_ ≃ 0.2 Å^−1^ [Fig. 6 ▸(*a*)], corresponding to the thicknesses of the Si slab of 30 Å. For sufficiently high *Q*_*z*,max_, a third reflection minimum can be observed at *Q*_*z*_ = 0.3 Å^−1^, which stems from the 20 Å-thick interstitial water layer. However, Fig. 6 ▸(*a*) indicates that extending the reflectivity to this value does not significantly increase the information gain further. Accordingly, the thicknesses of both sample layers are already well resolved when limiting the reflectivity to *Q*_*z*,max_ = 0.26 Å^−1^ (*t* = 6 h) (see Table 1 ▸, last column). This result is consistent with the canonical resolution estimate (Schalke & Lösche, 2000 ▸), which for *Q*_*z*,max_ = 0.26 Å^−1^ yields a smallest resolvable structure size of Δ*z* = π/*Q*_*z*,max_ = 12 Å. A discussion of the effect of a limited *Q*_*z*_ range on the information gain that goes beyond these rather qualitative arguments will require theory on time- and bandwidth-limited Gaussian channels (Cover & Thomas, 2006 ▸), and provides a promising avenue for future studies.

With respect to experimental design, it is useful to determine the critical value of *Q*_*z*, max_ for a particular sample to avoid spending neutron beamtime at unnecessarily high *Q*_*z*_ for which the signal-to-noise ratio becomes increasingly unfavorable. Real-world samples, as opposed to the simulated structures used in this work, do not necessarily have a smallest feature size that would define *Q*_*z*,max_. Therefore, future optimizations using more complex structural models with a larger range of feature sizes will be of high interest to determine how to limit the *Q*_*z*_ range of a measurement according to the smallest feature size of interest to the experimenter.

### 3.2. Influence of the substrate structure on information gain   

NR sample substrates are sometimes engineered to contain one or several nanoscopic layers of high SLD buried near the interface which are not part of the interfacial structure of interest. Magnetic reference layers that scatter incident neutrons differently, depending on the polarization of the neutron in a magnetic field, can be particularly powerful in elucidating interfacial details (Holt *et al.*, 2009 ▸). Such sample engineering has been demonstrated to allow for a direct inversion of reflectivity data in certain cases (Blasie *et al.*, 2003 ▸; Majkrzak *et al.*, 2009 ▸). Here we explore the effect of a buried nanoscopic layer on the information content of NR data using a previously published test case (Zimmermann *et al.*, 2000 ▸; Majkrzak & Berk, 2003 ▸).

**Figure 7:**
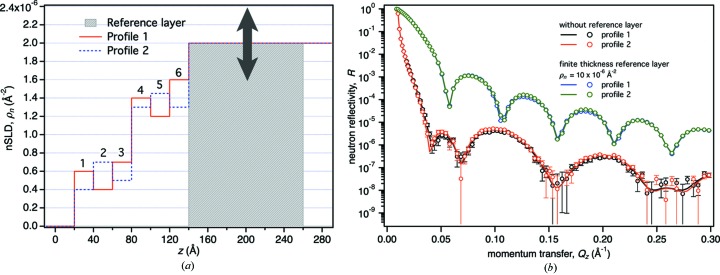
(*a*) Two distinct surface structures (profile 1 and profile 2) on an Si substrate in air are indistinguishable by (non-polarized) neutron reflection, as shown by the red and black reflectivity curves in (*b*) (Majkrzak & Berk, 2003 ▸). However, a reference layer buried beneath the surface structure [gray slab in (*a*) with 120 Å thickness and tunable SLD] is able to sufficiently increase the signal-to-noise ratio in the reflectivity to resolve the two profiles (blue and green curves, exemplarily shown for ρ_*n*_^nucl^ = 10 × 10^−6^ Å^−2^, ρ_*n*_^split^ = 0). Error bars indicate 68% confidence limits.

Zimmermann *et al.* (2000 ▸) described a set of distinct X-ray scattering length density profiles that yield nearly indistinguishable reflectivity curves. In turn, this prevents a unique determination of the SLD profile if any one of those reflectivities were measured. Majkrzak & Berk (2003 ▸) constructed a set of similar neutron SLD profiles that result in the same ambiguity (profiles 1 and 2 in Fig. 7 ▸). Both studies demonstrated that even partial knowledge of the sample structure can be insufficient to uniquely determine the SLD profile. It was shown that additional information, such as embedded reference structures, is necessary to uniquely determine the profiles.

Here, we systematically explore this problem by burying a tunable soft magnetic reference layer of finite thickness near the substrate surface [gray layer in Fig. 7 ▸(*a*)]. The SLD of the reference layer attains two values that depend on the polarization of the incident neutrons. The total SLD of the reference layer is the sum of the nuclear SLD and the magnetic splitting, ρ_*n*_^±^ = ρ_*n*_^nucl^ ± ρ_*n*_^split^. Both parameters depend on the choice of the magnetic material and were thus systematically varied in the analysis (see Table 3 ▸). For every point in the optimization space, including those with ρ_*n*_^split^ = 0, two reflectivity curves were simulated and analyzed, one for each neutron polarization. The statistical quality of each curve is equivalent to that obtained after 30 h counting on the Magik reflectometer at the NCNR. Reflectivities with ρ_*n*_^split^ = 0 are equivalent to those that would be obtained in a non-polarized NR experiment. The condition at which ρ_*n*_^nucl^ matches that of the underlying Si substrate and ρ_*n*_^split^ = 0 reproduces the structures described in the original work (Majkrzak & Berk, 2003 ▸).

**Figure 8:**
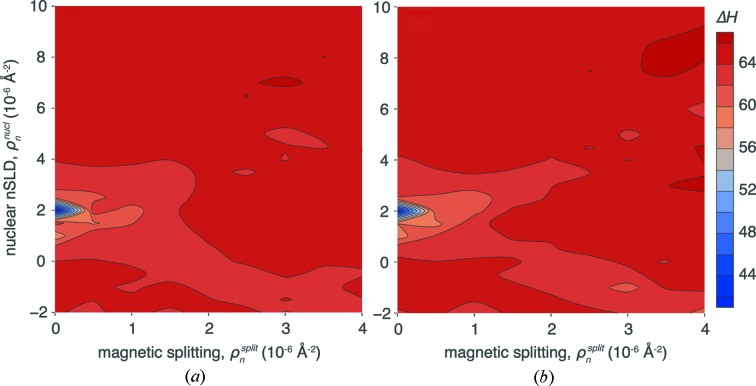
Information gain Δ*H* (KDN estimate) for profiles 1 (*a*) and 2 (*b*) as functions of the nuclear and magnetic SLDs of a buried reference layer (see Fig. 7 ▸).

The information gain Δ*H* from virtual NR measurements of profiles 1 and 2 as a function of ρ_*n*_^nucl^ and ρ_*n*_^split^ is shown in Fig. 8 ▸. In agreement with the previous work (Majkrzak & Berk, 2003 ▸), Δ*H* is small for the original configuration without reference layer (ρ_*n*_^nucl^ = 2 × 10^−6^ Å^−2^, ρ_*n*_^split^ = 0). Correlation plots between the fit parameters in this configuration (see Figs. S5 and S6 in the supporting information) reveal that the MCMC identifies several distinct solutions for the SLD values of layers 1–6 of the two interfacial structures shown in Fig. 7 ▸.

If one of the neutron-polarization-dependent SLDs of the reference layer is sufficiently different (by 2 × 10^−6^ Å^−2^ or more) from that of the substrate, a unique solution is obtained. This is reflected in Fig. 8 ▸ by an increase in information gain of ∼15 bits; and the parameter correlation plots (see supporting information) collapse into one solution. Most notably, a polarized NR experiment (ρ_*n*_^split^ ≠ 0) is not required to uniquely resolve profiles 1 and 2, as the information gain does not depend on ρ_*n*_^split^, as long as neither of ρ_*n*_^±^ matches that of the substrate. Table 3 ▸ lists the detailed results and their confidence limits for four selected points of the optimization: profile 1 without reference layer, profiles 1 and 2 with reference layer (ρ_*n*_^nucl^ = 6 × 10^−6^ Å^−2^, ρ_*n*_^split^ = 0), and profile 2 with reference layer and magnetic splitting (ρ_*n*_^nucl^ = 6 × 10^−6^ Å^−2^, ρ_*n*_^split^ = 2 × 10^−6^ Å^−2^). The information gain provided by the reference layer translates into significantly smaller uncertainties of the SLD values of the surface structure (layers 1–6).

In conclusion, a high-index reference layer boosts the overall reflectivity of the interfacial structure such that subtle details buried in noise of the reflectivity of the original structure become accessible. The near identity of the reflection spectra is not abrogated by the reference layer, but their overall magnitude is shifted to a level where the experiment can distinguish them, given the signal-to-noise ratio of a typical measurement.

## 4. Discussion   

We have implemented a framework based on Bayesian statistics and information theory (Liepe *et al.*, 2013 ▸) to optimize surface-sensitive scattering experiments by evaluating their information content as a function of experimental parameters. The information content 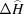 of the experiment is obtained by approximating the mutual information between the prior and posterior PDFs using virtual experiments. By necessity, we applied a number of restrictions to our implementation. Instead of computing the full mutual information, we use a single representative parameter vector θ from the prior distribution. We also simulated only up to ten data sets *y*(θ) per sample representation θ, which differ by Gaussian noise. Analyzing the information gain Δ*H* obtained from those *y*(θ), we showed that the observed standard deviation of Δ*H* is sufficiently small that its average can be used to approximate 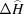.

We used two approaches to entropy calculation of the posterior PDF: the multivariate normal probability density approximation (MVN estimate) and an approach that samples directly from the posterior PDF (KDN estimate) (Kramer *et al.*, 2010 ▸). We observed that these approaches yield qualitatively consistent results, as exemplified in Figs. 3 ▸ and 4 ▸, in which the MVN approach tends to overestimate Δ*H*. On the other hand, the KDN algorithm produces occasional outliers that need to be eliminated.

Results on a simple reflectometry problem of a fluid-immersed sample (Fig. 2 ▸) demonstrated the usefulness of our approach. They confirm existing best practices on choosing the isotopic composition of aqueous solvents (Figs. 3 ▸ and 4 ▸). For this example, the largest gains in information were obtained when the scattering contrasts between different components of the sample structure – such as the substrate, the interfacial structure and the surrounding aqueous solvent – were large. The least gain in information was obtained when the SLD of the solvent was matched to that of the substrate and the interfacial structure. Under contrast-matching conditions, when zero reflectivity is measured, the computed information gain was larger than zero. Since the model contains implicit parameters, such as that of the substrate SLD, even measuring zero reflectivity provides information.

Our approach explicitly simulates background levels for different isotopic compositions of aqueous solvents and air. H_2_O-based solvents create a large incoherent background and yield the lowest signal-to-noise ratio in the measured reflectivity. Therefore, predominantly H_2_O-based solvents are at a disadvantage over D_2_O-based solvents with comparable scattering contrast to the substrate and interfacial structure. In Fig. 3 ▸, this effect contributes to an asymmetry in information gain around the minimum located at substrate-matching conditions (ρ_*n*_ = 2 × 10^−6^ Å^−2^). Similarly, Fig. 4 ▸ shows that a combination of CM4 (ρ_*n*_ = 4 × 10^−6^ Å^−2^) and D_2_O yields the same information gain as a combination of H_2_O and D_2_O, although the latter creates the greater overall scattering contrast and should, therefore, provide more information.

We showed for the same sample structure that an extension of the measurement time *t* per reflectivity curve above the empirically determined optimal value of 3–6 h on the simulated instrumentation is ineffective, as the information gain is a logarithmic function of *t* (Fig. 5 ▸). Furthermore, we determined the number of independent parameters supported by the data and the model by modeling the information gain as being limited by the Gaussian channel at the center of our optimization procedure (see Fig. 1 ▸). We neglected other losses of information that might occur during the calculation of the noise-free reflectivity *x*, and we relied on the assumption that the MCMC robustly finds the global solution of the fitting problem. In quantitative terms, the fit of the time dependence to equation (23) suggested that a measurement can resolve four independent parameters in this example. Equation (23) further allowed us to independently determine the rate of information gain as a function of measurement time per independent parameter. This constitutes an improvement over similar studies in small-angle scattering (Pedersen *et al.*, 2014 ▸).

We also studied the dependence of Δ*H* on the maximum momentum transfer *Q*_*z*,max_ in a measurement. Fig. 6 ▸ visualizes a result that is consistent with the rule of thumb expressed as the canonical resolution of a reflectivity experiment. For optimal information gain, a measurement must extend to a value of *Q*_*z*_ that depends on the smallest feature size of the sample. We showed for our example that extending the measurement beyond this value does not yield a significant increase in information gain, other than that stemming from the time spent measuring additional data points. Moreover, Fig. 6 ▸ demonstrates that the transition from ignorance to knowledge is rather sudden at this particular value of *Q*_*z*_ (left to right) – in contrast to the steady logarithmic increase in information gain as a function of simulated counting times (bottom to top).

While the virtual sample structure has a smallest length scale of 20 Å, real samples typically do not have such a limit. Nevertheless, the conclusion can be drawn that spending measurement time to assess reflectivities beyond the *Q*_*z*,max_ that is associated with the smallest feature size of interest to the experimenter is not advisable. These conclusions are drawn from the simple structure investigated here; but the clarity of the result indicates that the general approach will probably also yield valuable and interesting results for more complex systems. Here, we have not explicitly tested the main consequence of the Shannon–Nyquist sampling theorem – the notion that a minimum spacing between data points in *Q*_*z*_ is required to resolve a structure in real space with a limited total length (Vestergaard & Hansen, 2006 ▸; Shannon, 1949 ▸). However, this requirement is fulfilled in all virtual experiments simulated here; in fact, the simulated reflectivities are typically heavily oversampled.

While reference layers of high SLD and, in particular, magnetized reference layers probed with spin-polarized neutrons are increasingly used in NR investigations, details of how and to what degree they boost the information gain from the experiment have not been systematically investigated. Surprisingly, we found that performing a spin-polarized experiment is not required for maximum information gain in our example (see Fig. 8 ▸). Magnetic splitting of the reference layer SLD is only effective if the nuclear SLD is near that of the Si substrate. However, once the nuclear SLD is shifted away from ρ_*n*_^nucl^ = 2 × 10^−6^ Å^−2^, the information gain is largely independent of the splitting. Our results show essentially uniform information gain for 0 ≤ ρ_*n*_^split^ ≤ 4 × 10^−6^ Å^−2^, for sufficiently large ρ_*n*_^nucl^ > 4 × 10^−6^ Å^−2^. Fig. 7 ▸(*b*) visualizes the mechanism by which the nanoscopic reference layer increases the information gain. While the interference structure of the reflectivity curve from the sample with the reference layer is more regular than that without, showing how the high-SLD layer dominates the signal, the details of the interference pattern can be more precisely determined because the signal is raised above the noise. Therefore, profiles 1 and 2 can be distinguished on a sample with a reference layer, but not without such a layer.

Because spin-polarized measurements typically occur at 1/2 of the unpolarized beam intensity, we conclude that polarized reflectometry is not always effective for measurements such as those discussed here. It will be interesting to study how more complex samples, such as partially hydrated sample structures, are affected by reference layers. The result that a polarized neutron measurement is not required to resolve the sample structure does not contradict theoretical work that polarized reflectometry and magnetic reference layers are sufficient to analytically reconstruct the SLD profile of certain classes of samples (direct inversion) (Majkrzak *et al.*, 2009 ▸), nor that analytical data inversion recovers the maximum information content from the experimental data (phase-inversion principle) (Berk & Majkrzak, 2009 ▸).

## 5. Conclusion   

We implemented a Bayesian and information theoretical framework to determine the information gain from reflectometry experiments with the purpose of experimental optimization. We applied this framework to a selection of test problems, demonstrating its usability and confirming many best practices that have guided the design of reflectometry experiments for a long time. At the same time, we gained non-intuitive insights that challenge some of them. A next step in this development will be an extension to more complex, and more relevant, applications of reflectometry in current research. Marginalization of the posterior PDF will be required to tailor the experiment effectively to a subset of parameters that are of immediate interest to the experimenter. With this in place, we predict significant utility of this framework for the optimization of reflectometry experiments from complex biomimetic interfaces.

## Supplementary Material

Details regarding the simulation of reflectivity data and additional figures. DOI: 10.1107/S1600576718017016/ge5055sup1.pdf

## Figures and Tables

**Table 1:** Simulation parameters and MCMC fit results for a virtual NR measurement of the system shown in Fig. 2 ▸ in which one bulk solvent SLD was optimized Where ranges are given in the first column, the parameter was systematically varied within these boundaries. Median parameter values and 68% confidence limits were determined by an MCMC fit of the simulated data and are given for selected solvent SLD values of the entire optimization range shown in Fig. 3 ▸.

			MCMC fit results (θ | *y*)			
			Solvent SLD, ρ_*n*_ (10^−6^ Å^−2^)			
Model parameter	Parameterized sample representation, θ	Fit boundaries, prior PDF limits	−0.5	2	6.5	
Thickness of interstitial water (Å)	20	±10	21 ± 1	20 ± 7		20.0 ± 0.2
Thickness of porous Si layer (Å)	30	±10	29.1 ± 0.9	29 ± 7		29.8 ± 0.2
SLD of porous Si layer (10^−6^ Å^−2^)	2.07	±1	2.0 ± 0.1	2.1 ± 0.1		2.1 ± 0.2
Volume fraction of porous Si layer	0.95	±0.05	0.95 ± 0.04	0.95 ± 0.03		0.95 ± 0.04
SLD of solvent, *ρ_n_* (10^−6^ Å^−2^)	[−0.5, 6.5]	±0.5	−0.50 ± 0.03	2.1 ± 0.1		6.50 ± 0.01
Interfacial roughness (Å)	3	±1	2.9 ± 0.6	3.0 ± 0.7		3.4 ± 0.5
Log_10_ of background†	−8	±1	−7.9 ± 0.7	−8.1 ± 0.6		−8.0 ± 0.7

† A constant background is routinely fitted as a free parameter to each experimental NR curve, and the same procedure is adopted here. This parameter accounts for insufficient background subtraction during data reduction and is a small fraction of the total background (see supporting information).

**Table 2:** Results for a virtual NR experiment on the system shown in Fig. 2 ▸, which allows for two measurements with two distinct bulk solvent SLDs that were optimized (Fig. 4 ▸) (for other details refer to Table 1 ▸)

			MCMC fit results (θ | *y*)		
			Solvent SLDs ρ_*n*_^1^, ρ_*n*_^2^ (10^−6^ Å^−2^)		
Model parameter	Parameterized sample representation, θ	Fit boundaries, prior PDF limits	(2.0, 2.0)	(−0.5, 6.5)	(4.0, 6.5)
Thickness of interstitial water (Å)	20	±10	21 ± 8	19.9 ± 0.2	19.9 ± 0.2
Thickness of porous Si layer (Å)	30	±10	29 ± 7	30.0 ± 0.1	30.2 ± 0.2
SLD of porous Si layer (10^−6^ Å^−2^)	2.07	±1	2.1 ± 0.1	2.08 ± 0.03	2.15 ± 0.06
Volume fraction of porous Si layer	0.95	±0.05	0.95 ± 0.03	0.955 ± 0.009	0.97 ± 0.02
SLD of solvent 1, ρ_*n*_^1^ (10^−6^ Å^−2^)	[−0.5, 6.5]	±0.5	2.1 ± 0.1	6.499 ± 0.004	6.493 ± 0.003
SLD of solvent 2, ρ_*n*_^2^ (10^−6^ Å^−2^)	[−0.5, 6.5]	±0.5	2.1 ± 0.1	−0.49 ± 0.02	4.001 ± 0.002
Interfacial roughness (Å)	3	±1	3.0 ± 0.7	2.7 ± 0.5	3.6 ± 0.4
Log_10_ of background 1	−8	±1	−8.0 ± 0.7	−7.9 ± 0.7	−8.2 ± 0.5
Log_10_ of background 2	−8	±1	−7.7 ± 0.7	−8.0 ± 0.7	−7.9 ± 0.7

**Table 3:** Results for a virtual reflectivity experiment with polarized neutrons on the system shown in Fig. 7 ▸(*a*) A 120 Å-thick reference layer is buried beneath the interfacial profile of interest. The SLD of this layer consists of a nuclear part and a magnetic splitting which leads to distinct SLD values seen by different neutron polarizations in a scattering experiment. The impact of (ρ_*n*_^nucl^, ρ_*n*_^split^) on Δ*H* was systematically evaluated in this analysis and is presented here for selected values. For other details refer to Table 1 ▸.

			MCMC fit results (θ | *y*)			
			SLD of reference layer, (ρ_*n*_^nucl^, ρ_*n*_^split^) (10^−6^ Å^−2^)			
			Profile 1		Profile 2	
Parameter	Parameterized sample representation, θ	Fit boundaries, prior PDF limits	(2, 0)	(6, 0)	(6, 0)	(6, 2)
Nuclear SLD reference layer, ρ_*n*_^nucl^ (10^−6^ Å^−2^)	[−2, 10]	±0.5	2.000 ± 0.002	6.002 ± 0.002	5.997 ± 0.002	6.002 ± 0.002
Magnetic SLD of reference layer, ρ_*n*_^split^ (10^−6^ Å^−2^)	[0, 4]	±0.5	0.000 ± 0.004	0.001 ± 0.002	0.001 ± 0.002	2.001 ± 0.002
SLD layer 1 (10^−6^ Å^−2^)	0.6/0.4	[−0.5, 2.5]	0.5 ± 0.2	0.601 ± 0.006	0.402 ± 0.006	0.400 ± 0.006
SLD layer 2 (10^−6^ Å^−2^)	0.4/0.7	[−0.5, 2.5]	0.6 ± 0.1	0.407 ± 0.005	0.701 ± 0.006	0.698 ± 0.006
SLD layer 3 (10^−6^ Å^−2^)	0.7/0.5	[−0.5, 2.5]	0.6 ± 0.2	0.703 ± 0.006	0.493 ± 0.006	0.509 ± 0.006
SLD layer 4 (10^−6^ Å^−2^)	1.4/1.3	[−0.5, 2.5]	1.4 ± 0.2	1.400 ± 0.006	1.297 ± 0.006	1.302 ± 0.005
SLD layer 5 (10^−6^ Å^−2^)	1.2/1.45	[−0.5, 2.5]	1.4 ± 0.1	1.197 ± 0.006	1.451 ± 0.005	1.437 ± 0.005
SLD layer 6 (10^−6^ Å^−2^)	1.6/1.3	[−0.5, 2.5]	1.6 ± 0.2	1.602 ± 0.006	1.304 ± 0.006	1.298 ± 0.006
Interfacial roughness (Å)	3	[2, 5]	2.7 ± 0.5	3.0 ± 0.1	2.9 ± 0.1	3.09 ± 0.08
Log_10_ background spin ↑↑	−8	[−9, −5]	−8.1 ± 0.2	−8.3 ± 0.4	−8.3 ± 0.4	−7.9 ± 0.2
Log_10_ background spin ↓↓	−8	[−9, −5]	−8.0 ± 0.1	−7.7 ± 0.3	−8.1 ± 0.5	−7.7 ± 0.4

## Footnotes

1 With current technology, the computation of *p*(θ | *y*) for a model of average complexity used in this work takes approximately 1.5 CPU hours. The calculation of *H*(Θ | *y*) is negligible in comparison. With a sample size of 1000 in *Y*, a nested Monte Carlo simulation would average 1500 CPU hours ≃ 60 CPU days per . Optimizations in this work would require the calculation of between 15 and 200 values of , requiring 900 to 12 000 CPU days total.

2 This neglects the diminishing effect of background on the relative variance. However, the background arising from the D_2_O solvent in the current sample is sufficiently small.
