# Supplementary material for: Optimization of reflectometry experiments using information theory
Source: J Appl Crystallogr. 2019 Feb 1;52(Pt 1):47–59. doi: 10.1107/S1600576718017016 (PMC6362612; doi:10.1107/S1600576718017016)
Supplement: Supplementary file 1 [file j-52-00047-sup1.pdf]

JOURNAL OF
APPLIED
CRYSTALLOGRAPHY

Volume 52 (2019)

Supporting information for article:

Optimization of Reflectometry Experiments using Information Theory

Bradley W. Treece, Paul A. Kienzle, David P. Hoogerheide, Charles F. Majkrzak, Mathias Lösche and Frank Heinrich

Supporting information

Authors

Bradley W. Treece[a], Paul A. Kienzle[b], David P. Hoogerheide[b], Charles F. Majkrzak[b], Mathias Lösche[abc] and Frank Heinrich[ab]*

[a]Department of Physics, Carnegie Mellon University, 5000 Forbes Avenue, Pittsburgh, Pennsylvania, 15213, United States

[b]Center for Neutron Research, National Institute of Standards and Technology, 100 Bureau Drive, Gaithersburg, Maryland, 20899-6102, United States

[c]Department of Biomedical Engineering, Carnegie Mellon University, 5000 Forbes Avenue, Pittsburgh, Pennsylvania, 15213, United States

Correspondence email: fheinrich@cmu.edu

S1. Calculation of Reflectivity Data and Simulation of Error Bars

Error bars on reflectivity curves are calculated from a noise-free simulated reflectivity $X_{S,E}(\theta) \rightarrow x(Q_z) = R_s(Q_z)$ under the assumption of typical measurement parameters from previous neutron reflectometry experiments using the NCNR wet cell (Heinrich & Lösche, 2014) at the Magik reflectometer at the NIST Center for Neutron Research (NCNR). We only consider uncertainties associated with the specular reflectivity and the background measurement as significant contributors to the total measurement uncertainty. Uncertainties stemming from the normalization of the reflectivity by the separately measured incident beam intensity, and by the inline beam monitor, are neglected.

The total number of detector counts for the specular reflectivity $n_s(Q_z)$ at any given data point with momentum transfer Q_z (from here on denoted as q for brevity) is a product of the beam-defining aperture openings before the sample $s_1(q)$ and $s_2(q)$, the measurement time $t(q)$, the value of the specular reflectivity $R_s(q)$, and the flux of the neutron beam i is given as a time and aperture normalized neutron count. Beam height, instrument geometry, and wavelength spread are implicitly contained in this variable. For $n_s(q)$ to be proportional to the product $s_1(q)s_2(q)$ requires sufficient divergence and homogeneity of the beam within the scattering plane.

$$n_s(q) = i\, R_s(q) s_1(q) s_2(q) t(q)$$

During a reflectivity measurement, with increasing q, the cross-section of the beam intersecting the sample plane (beam footprint) is kept constant and the measurement time is increased. Both measures

compensate for the decrease of $R_s(q)$ with increasing q. Specifically, we have implemented the following q-dependence.

$$s_{1,2}(q) = c_{1,2}q$$
$$t(q) = c_3 + c_4q^2$$

The number of counts $n_b(q)$ measured for the background at the specular position is equally approximated the following equation.

$$n_b(q) = i\ c_{b,mat}s_1(q)s_2(q)t(q)$$

The constant $c_{b,mat}$ is a property of the material in the neutron beam that produces the background radiation. Here, it is assumed to be independent of q, which is not strictly justified. A better approximation must account for the momentum transfer q, the neutron wavelength λ ,the thickness of the fluid reservoir D, and the inverse free path length ϵ of the neutron in the reservoir medium before absorption occurs (David Hoogerheide, Frank Heinrich, Brian Maranville and Charles Majkrzak, unpublished data):

$$c_{b,mat} \propto \frac{q\lambda}{8\pi\epsilon}\left(1 - e^{-\frac{8\pi\epsilon D}{q\lambda}}\right)$$

Apertures after the sample do not contribute significantly to the specular reflectivity, nor the background, because they are generally large enough to capture the entire volume of illuminated sample.

The background corrected reflectivity, which is not normalized by the incident beam is:

$$R = (n_s + n_b) - n_b$$

The relative uncertainty on this reflectivity following Poisson statistics is given by the following equation. Under the assumption of negligible contributions from beam normalization and beam monitor normalization this uncertainty will be directly applied to the simulated reflectivity $R_s(q)$.

$$\frac{dR}{R} = \frac{\sqrt{(n_s + n_b) + n_b}}{n_s}$$

It remains to determine all constants $c_{1\ldots4}$, $c_{b,mat}$, and i. Constants $c_{1\ldots4}$ and i are calculated from the neutron count rate of a typical scan trajectory measured in D_2O (see Table S1). At $Q_{z,min}$ = 0.008 Å^{-1} the intensity factor i can be determined, because the momentum transfer is below the critical value below which total reflection of the beam from the sample occurs.

Table S1 Table S1: Typical instrument settings and experimental counts for a 3h-scan using a NCNR wet cell filled with D_2O at the Magik reflectometer. The beam footprint on the sample is kept constant during the measurement at 2.5 cm x 5 cm.

Momentum transfer value:	$Q_{z,min}$ = 0.008 Å^{-1}	$Q_{z,max}$ = 0.325 Å^{-1}
Detector counts, n	11809	658
Monitor counts	9817	2,890,340
Aperture 1 opening, s_1	0.108	4.397
Aperture 2 opening, s_2	0.108	4.397
Aperture 3 opening, s_3	3.430	11.754
Aperture 4 opening, s_4	3.430	11.754
Measurement time per point, t	18	208

The background scattering cross-section $c_{b,mat}$ could be determined in the same way from representative experimental data. However, it is convenient to rather determine $c_{b,mat}$ by defining at which level of reflectivity the background count number equals that of the specular count number. It can be seen that within the current description that $c_{b,mat}$ assumes exactly this reflectivity value.

$$n_s = n_b$$
$$i\, R_s(q)\, s_1(q)\, s_2(q)\, t(q) = i\, c_{b,mat}\, s_1(q)\, s_2(q)\, t(q)$$
$$R_s(q) = c_{b,mat}$$

For the above given example, the obtained values are summarized in Table S2. The typical runtime of the specular and background scans on NG-D Magik at the NCNR is about 3h for a single sweep of the entire Q_z-range, and 6h for a standard measurement comprising two sweeps. If not stated otherwise, in this work, reflectivity curves of 6h total counting time are simulated. Simulated experiments containing more than one reflectivity measurement, have an accordingly longer total counting time.

Table S2 Table S2: Constants obtained for a typical NR measurement using the NCNR wet cell.

Constant	Value
c_1 (mm Å)	13.529
c_2 (mm Å)	13.529
c_3 (s)	17.885
c_4 (s Å^2)	1799.91

i (s^{-1} mm^{-2})	56246.19
$c_{b,mat}$	1.10×10^{-5} (H_2O)<br>1.25×10^{-6} (D_2O)<br>1.00×10^{-7} (air)

The following figures compare experimental and simulated measurement uncertainties, showing sufficiently good agreement.

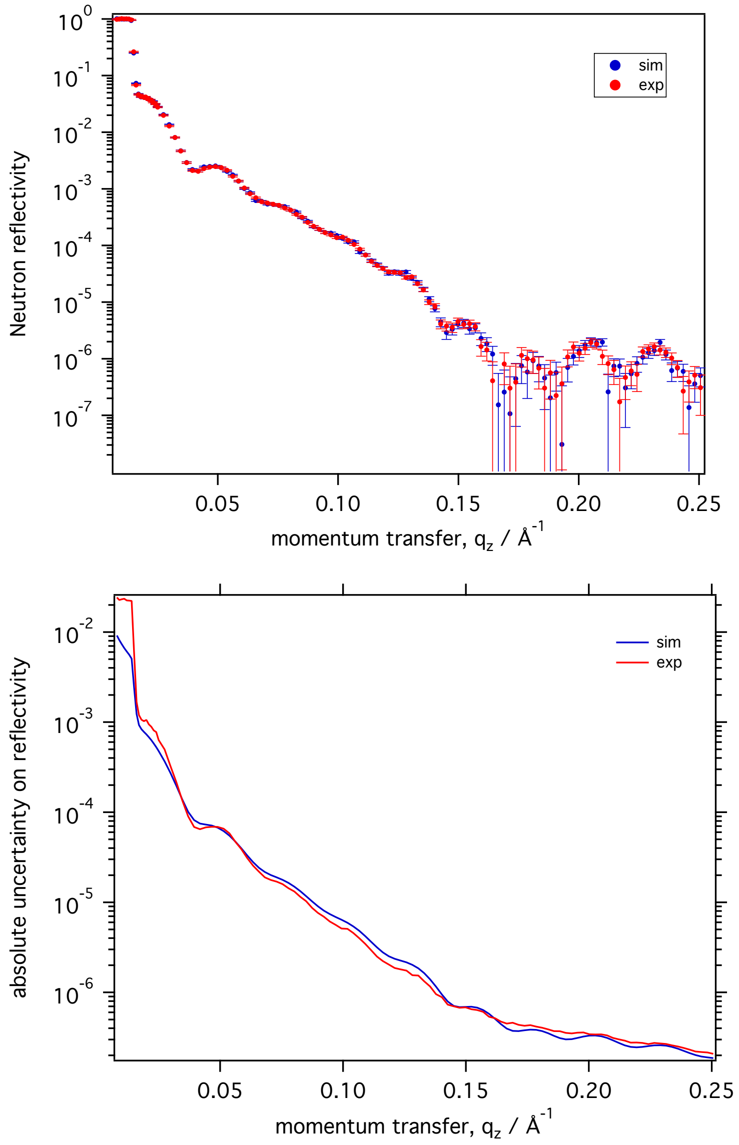

Figure S1 (Top) Simulated and experimental reflectivities of a sparsely tethered lipid bilayer membrane on a gold-coated silicon wafer in a D_2O-based bulk solvent (Heinrich & Lösche, 2014). (Bottom) Absolute values of the experimental and simulated error bars for the stBLM in D_2O.

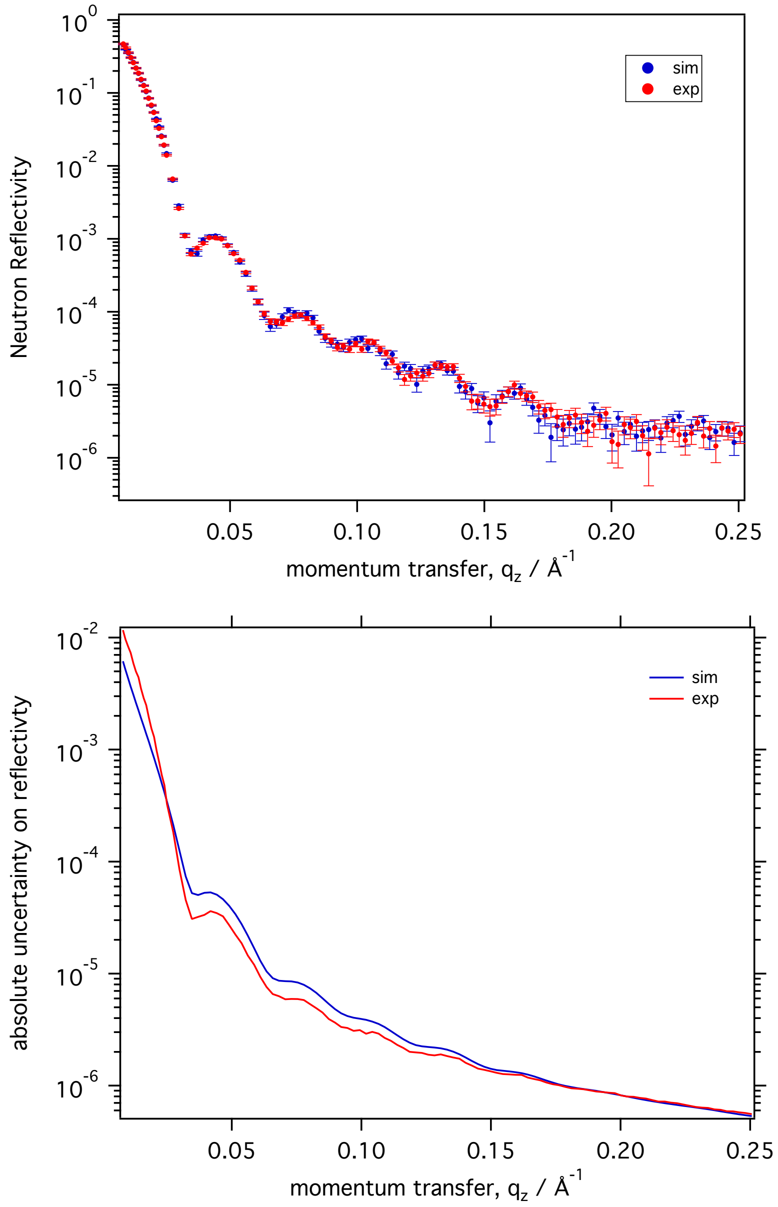

Figure S2 (Top) Simulated and experimental reflectivities of a sparsely tethered lipid bilayer membrane on a gold-coated silicon wafer in an H_2O-based bulk solvent. (Bottom) Absolute values of the experimental and simulated error bars for the stBLM in D_2O.

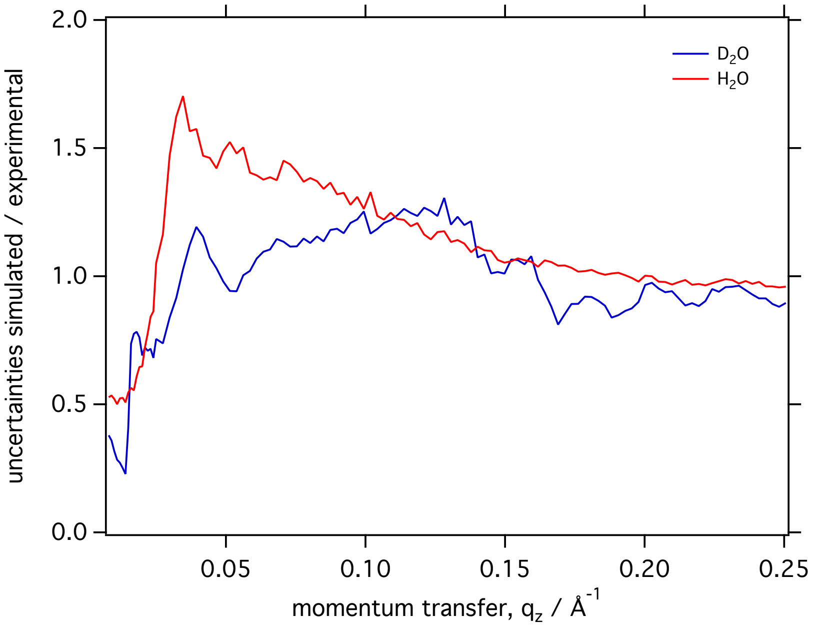

Figure S3 The ratio of simulated and experimental error bars for the D_2O and the H_2O contrast of the stBLM reflectivity.

S2. Results

S2.1. Counting Time

Figure S4 validates that the uncertainty dR/R on the simulated reflectivity data points as a function of counting time t follows Poisson statistics.

$$\frac{dR}{R} \propto t^{-0.5}$$

In Figure 5 and the main text we show that the information gain ΔH follows a different dependence on counting time t for this example.

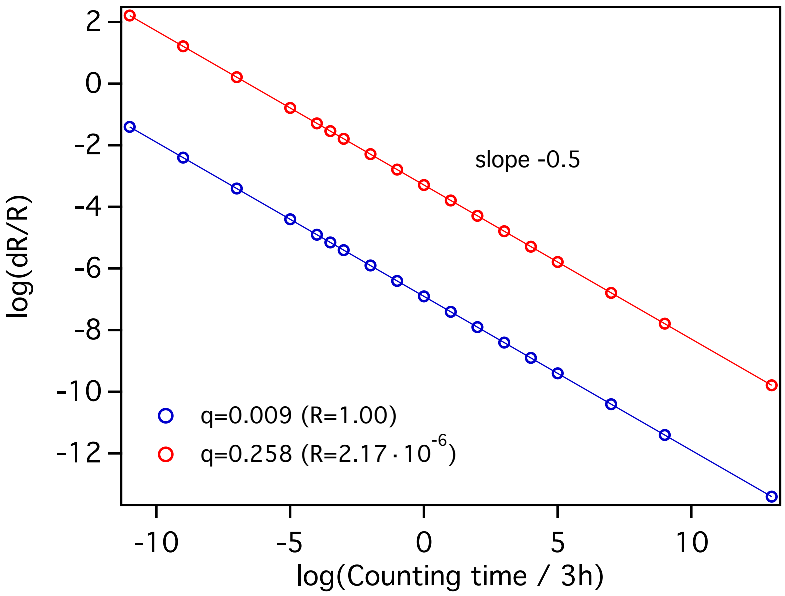

Figure S4 dR/R for the first and the last data point in the simulated reflectivity as a function of overall counting time t for the entire reflectivity curve measured with D_2O solvent shown in Figure 2B (individual counting times per data point depend on Q_z, see Table S1). The first data point at Q_z=0.009 Å^{-1} is below the critical angle for D_2O against silicon and there is no significant background. At the highest momentum transfer of Q_z=0.258 Å^{-1} the background causes a consistently higher dR/R. The log-log plot yields a slope of -0.5, in agreement with the Poisson counting statistics.

S2.2. Influence of the Substrate Structure on the Information Gain

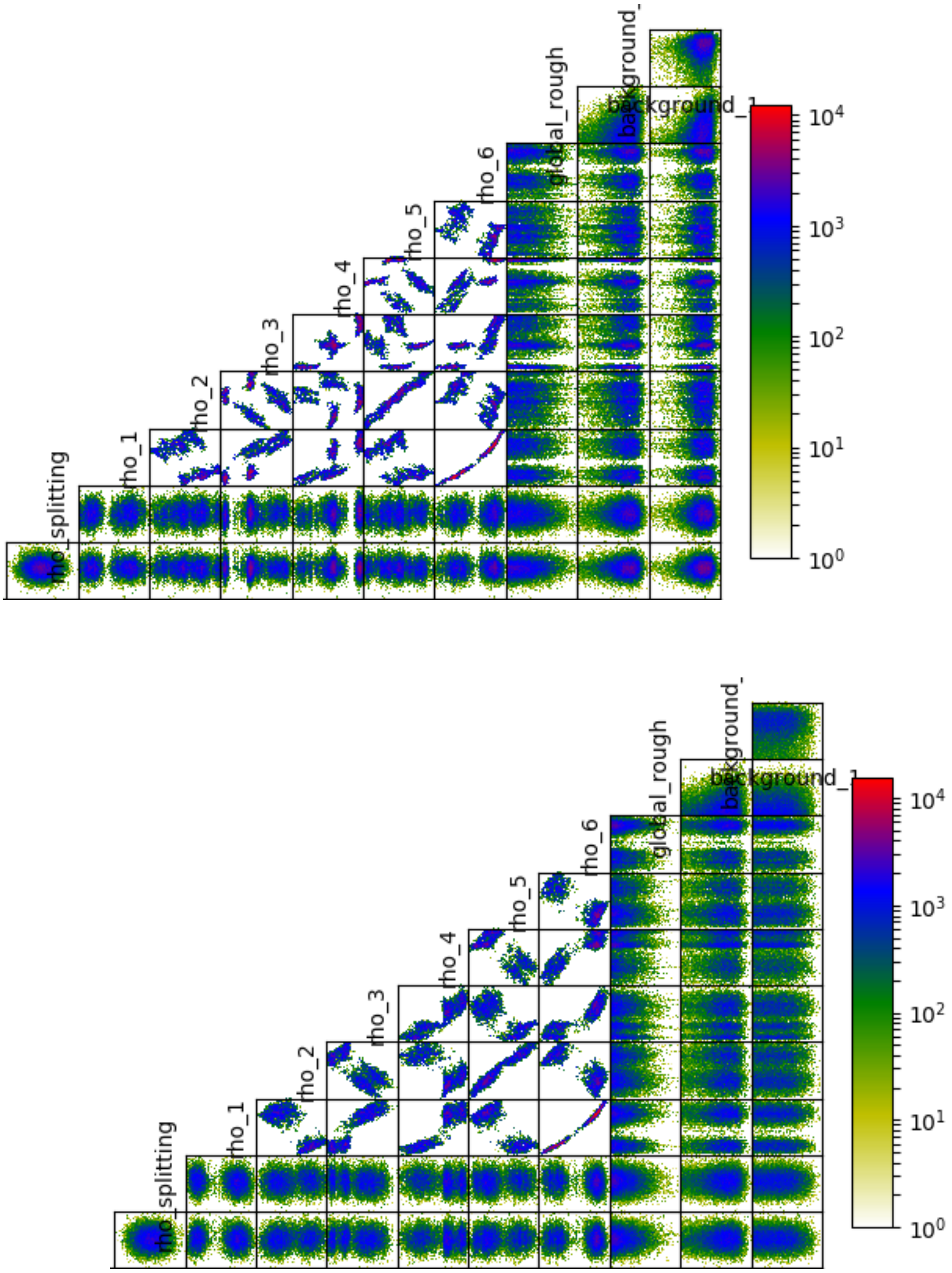

Figure S5 Fit parameter correlations for profile 1 (top) and profile 2 (bottom) for a reference layer with a nuclear SLD of 2×10^{-6} Å^{-2} and zero magnetic splitting (see Figure 7). Multiple distinct solutions are clearly visible for the SLD values of layers 1 to 6.

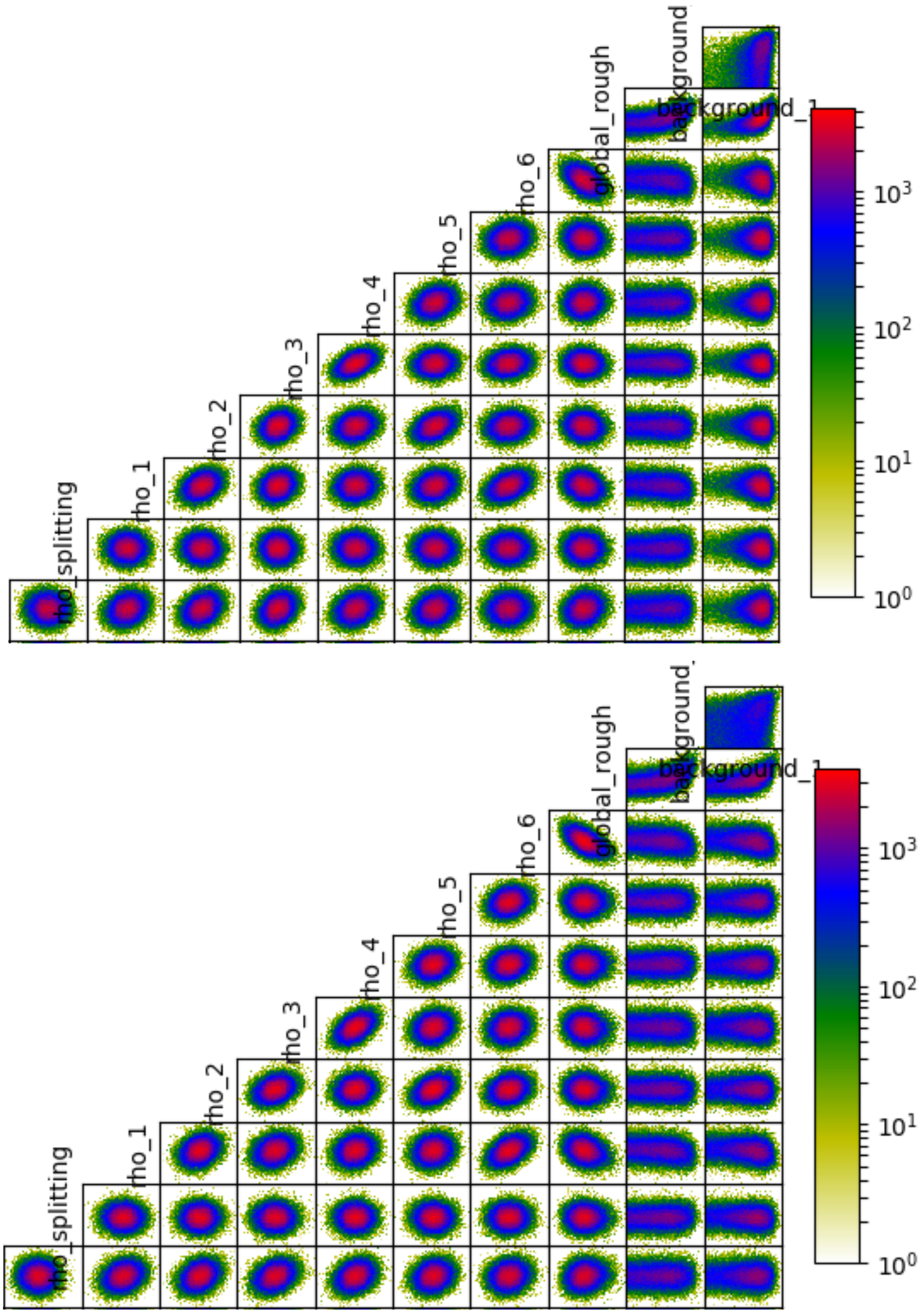

Figure S6 Fit parameter correlation for profile 1 (top) and profile 2 (bottom) for a reference layer with a nuclear SLD of 6×10^{-6} Å^{-2} and zero magnetic splitting (see Figure 7). Only a single solution is observed for the SLD values of layers 1 to 6.
